# Supplementary material for: Predictors of school students’ leisure-time physical activity: An extended trans-contextual model using Bayesian path analysis
Source: PLoS One. 2021 Nov 12;16(11):e0258829. doi: 10.1371/journal.pone.0258829 (PMC8589162; doi:10.1371/journal.pone.0258829)
Supplement: S1 File — (DOCX) [file pone.0258829.s001.docx]

**Appendix A.** Details of Measures Used in the Extended Trans-Contextual Model.

| Measure | Subscale (if applicable) | Items | Scale (if applicable) |
| --- | --- | --- | --- |
| Perceived autonomy support from PE teacher |  | My PE teacher tries to understand how I see things before suggesting a new way to do things  I feel that my PE teacher provides me with choices, options, and suggestions about whether to do physical activity  My PE teacher displays conﬁdence in my ability to do physical activity  My PE teacher encourages me to do physical activity  My PE teacher listens to me about my physical activity  I am able to talk to my PE teacher about physical activity  My PE teacher cares about the physical activity I do  I feel that my PE teacher provides me with choices, options, and suggestions about whether to do physical activity in my free time  My PE teacher displays conﬁdence in my ability to do physical activity in my free time  My PE teacher encourages me to do physical activity in my free time  My PE teacher listens to me about my physical activity in my free time  I am able to talk to my PE teacher about physical activity in my free time  My PE teacher cares about the physical activity I do in my free time | 1 = Strongly disagree,  7 = Strongly agree |
| Autonomous motivation (PE) | Intrinsic motivation | Stem: I do PE…  … Because I enjoy PE  … Because PE is fun | 1 = Not true for me,  7 = Very true for me |
|  | Identified regulation | … Because it is important to me to do well in PE  … Because it is important to me to improve in PE | 1 = Not true for me,  7 = Very true for me |
| Autonomous motivation (leisure time) | Intrinsic motivation | Stem: I do physical activity during my free time…  … Because I enjoy doing physical activity  … Because it is fun | 1 = Not true for me,  7 = Very true for me |
|  | Identified regulation | … Because I value the benefits of physical activity  … Because it’s important to me to do physical activities | 1 = Not true for me,  7 = Very true for me |
| Intention |  | I intend to do active sports and/or vigorous physical activities during my leisure time in the next 5 weeks  I plan to do active sports and/or vigorous physical activities during my leisure time in the next 5 weeks | 1 = Strongly disagree,  7 = Strongly agree |
| Attitudes |  | Stem: Participating in active sports and/or vigorous physical activities during my leisure time in the next 5 weeks is… | 1 = Unenjoyable, 7 = Enjoyable;  1 = Bad, 7 = Good;  1 = Useless, 7 = Useful |
| Subjective norm |  | Most people who are important to me think I should do active sports and/or vigorous physical activities during my leisure time for the next 5 weeks  Most people important to me put pressure on me to do active sports and/or vigorous physical activities during my leisure time for the next 5 weeks | 1 = Strongly disagree,  7 = Strongly agree |
| Perceived behavioral control |  | How much control do you have over doing active sports and/or vigorous physical activities in your leisure time in the next 5 weeks?  I am confident I could do active sports and/or vigorous physical activities during  my leisure time in the next 5 weeks | 1 = Very little control,  7 = Complete control;  1 = Strongly disagree,  7 = Strongly agree |
| Habit | Automaticity | Physical activity is something I do automatically  Physical activity is something I do without having to consciously remember  Physical activity is something I do without thinking  Physical activity is something I start doing before I realize I’m doing it | 1 = Completely uncertain,  7 = Completely certain |
| Trait self-control |  | I have difficulty starting tasks (reversed)  I get my chores done right away  I find it difficult to get down to work (reversed)  I am always prepared  I often waste my time (reversed)  I start tasks right away  I tend to postpone decisions (reversed)  I like to get to work at once  I need a push to get started (reversed)  I tend to carry out my plans | 1 = Not like me at all;  4 = Very much like me |
| Physical activity participation |  | During the last 7 days, on how many days did you do vigorous physical activities like heavy lifting, heavier garden or construction work, chopping wood, aerobics, jogging/running or fast bicycling?  Think only about those physical activities that you did for at least 10 minutes at a time.  During the last 7 days, on how many days did you do moderate physical activities like gardening, cleaning, bicycling at a regular pace, swimming or other fitness activities.  Think only about those physical activities that you did for at least 10 minutes at a time. Do not include walking.  Think about the time you spent walking in the last 7 days. This includes at work and at home, walking to travel from place to place, and any other walking that you might do solely for recreation, sport, exercise, or leisure.  During the last 7 days, on how many days did you walk for at least 10 minutes at a time? | _____days;  How much time did you usually spend doing vigorous/moderate/walking/ (physical activities) on one of those days?;  ____ hours ___ minutes |

**Appendix B.** Processing of International Physical Activity Questionnaire (IPAQ) Data.

Data from the IPAQ were used to develop a composite measure of physical activity participation based on recommended guidelines (Craig et al., 2003; The IPAQ Group, 2012). Initially, the values from hours or hours and minutes were translated into minutes. Values of 15, 30, 45, 60, or 90 in the “hours” column of the questionnaire were transferred to the “minutes” column. Participants who made mistakes not addressed in the guidelines (e.g., value of 25 hours or missing data for days or time) were excluded. Secondly, when the sum of weekly PA exceeded 6720 minutes per week (i.e., 16 hours × 60 minutes × 7 days) participants were excluded with the assumption that they were sleeping eight hours a day. Thirdly, truncation (re-coding) of IPAQ scores was performed. Any given activity above three hours was re-coded to three hours (i.e., 180 minutes), permitting a maximum value of 21 hours per activity (three hours × seven days) and 63 hours (i.e., 3780 minutes) of total PA per week. Similarly, any value below 10 minutes was recoded to zero. Finally, due to the skewed distributions associated with the IPAQ, the MET-minutes per week were log transformed using the log10 + 1 transformation in SPSS to account for zero values. The distribution of the log-transformed MET-minutes has the property of being more normally distributed, in contrast to the raw IPAQ scores (Rzewnicki, Auweele, & Bourdeaudhuij, 2003).

**References**

Craig, C. L., Marshall, A. L., Sjostrom, M., Bauman, A. E., Booth, M. L., Ainsworth, B. E., . . . Oja, P. (2003). International physical activity questionnaire: 12-country reliability and validity. *Medicine and Science in Sports and Exercise, 35*, 1381-1395. https://doi.org/10.1249/01.mss.0000078924.61453.fb

Rzewnicki, R., Auweele, Y. V., & Bourdeaudhuij, I. D. (2003). Addressing overreporting on the International Physical Activity Questionnaire (IPAQ) telephone survey with a population sample. *Public Health Nutrition, 6*, 299-305. https://doi.org/10.1079/PHN2002427

The IPAQ group. (2012). International Physical Activity Questionnaire. Retrieved March 1, 2012, from http://www.ipaq.ki.se/

Appendix C

*Descriptive Statistics and Zero-Order Intercorrelations for the Extended Trans-Contextual Model Constructs, Behavior Measure, and Socio-Demographic and Control Variables*

| Variable | ω | *M* | SD | 1 | 2 | 3 | 4 | 5 | 6 | 7 | 8 | 9 | 10 | 11 | 12 | 13 | 14 |
| --- | --- | --- | --- | --- | --- | --- | --- | --- | --- | --- | --- | --- | --- | --- | --- | --- | --- |
| 1. Perceived autonomy support (PE) | .912 | 5.681 | 0.867 | – |  |  |  |  |  |  |  |  |  |  |  |  |  |
| 2. Autonomous motivation (PE) | .888 | 5.647 | 1.314 | .447^***^ | – |  |  |  |  |  |  |  |  |  |  |  |  |
| 3. Autonomous motivation (LT) | .921 | 5.876 | 1.253 | .391^***^ | .614^***^ | – |  |  |  |  |  |  |  |  |  |  |  |
| 4. Intention | .890 | 5.651 | 1.282 | .294^***^ | .479^***^ | .733^***^ | – |  |  |  |  |  |  |  |  |  |  |
| 5. Attitude | .805 | 6.070 | 1.006 | .300^***^ | .393^***^ | .582^***^ | .591^***^ | – |  |  |  |  |  |  |  |  |  |
| 6. Subjective norms | .815 | 4.769 | 1.451 | .106 | .204^***^ | .189^**^ | .315^***^ | .189^**^ | – |  |  |  |  |  |  |  |  |
| 7. PBC | .756 | 6.030 | 1.018 | .262^***^ | .253^***^ | .391^***^ | .576^***^ | .429^***^ | .175^**^ | – |  |  |  |  |  |  |  |
| 8. Habit | .851 | 5.127 | 1.373 | .262^***^ | .472^***^ | .650^***^ | .566^***^ | .402^***^ | .162^**^ | .360^***^ | – |  |  |  |  |  |  |
| 9. Self-control | .875 | 2.914 | 0.542 | .316^***^ | .496^***^ | .478^***^ | .477^***^ | .345^***^ | .141^*^ | .324^***^ | .454^***^ | – |  |  |  |  |  |
| 10. Past physical activity behavior | .912 | 3.696 | 0.373 | .151^*^ | .254^***^ | .524^***^ | .507^***^ | .335^***^ | .167^**^ | .368^***^ | .492^***^ | .284^***^ | – |  |  |  |  |
| 11 Physical activity | .888 | 3.604 | 0.470 | .102 | .229^***^ | .355^***^ | .351^***^ | .342^***^ | .121^*^ | .206^**^ | .313^***^ | .282^***^ | .575^***^ | – |  |  |  |
| 12. Intervention | – | – | – | -.141^*^ | .067 | -.061 | -.046 | -.124^*^ | -.024 | -.049 | .023 | .012 | .066 | .052 | – |  |  |
| 13. Gender | – | – | – | .086 | -.103 | .003 | .006 | .099 | -.042 | -.073 | -.149^*^ | -.087 | -.151^**^ | -.069 | -.007 | – |  |
| 14. Age | – | 14.506 | 0.704 | .051 | .007 | .056 | .085 | .098 | .033 | .085 | .009 | -.067 | .002 | -.042 | -.237^***^ | .066 | – |

*Note*. PE = Physical education context; LT = Leisure-time context; PBC = Perceived behavioral control; Past physical activity behavior = Past leisure-time physical activity behavior; Physical activity = Self-reported leisure-time physical activity participation.

^*^*p* < .05 ^**^*p* < .01 ^***^*p* < .001.
